# Scutellarin alleviates microglia‐mediated neuroinflammation and apoptosis after ischemic stroke through the PI3K/AKT/GSK3*β* signaling pathway

**DOI:** 10.1002/ccs3.12023

**Published:** 2024-04-12

**Authors:** Zhaoda Duan, Haolun Chen, Wei Miao, Jing He, Dongyao Xu, Zhi Qi, Li Yang, Wenji Jia, Chunyun Wu

**Affiliations:** ^1^ Department of Anatomy and Histology/Embryology Faculty of Basic Medical Sciences Kunming Medical University Kunming China; ^2^ Department of Neurology The Second Affiliated Hospital Kunming Medical University Kunming China

**Keywords:** ischemic stroke, microglia, network pharmacology, PI3K/AKT/GSK3*β*, scutellarin

## Abstract

Microglia are resident immune cells in the central nervous system that are rapidly activated to mediate neuroinflammation and apoptosis, thereby aggravating brain tissue damage after ischemic stroke (IS). Although scutellarin has a specific therapeutic effect on IS, the potential target mechanism of its treatment has not been fully elucidated. In this study, we explored the potential mechanism of scutellarin in treating IS using network pharmacology. Lipopolysaccharide (LPS) was used to induce an in vitro BV‐2 microglial cell model, while middle cerebral artery occlusion (MCAO) was used to induce an in vivo animal model. Our findings indicated that scutellarin promoted the recovery of cerebral blood flow in MCAO rats at 3 days, significantly different from that in the MCAO group. Western blotting and immunofluorescence revealed that scutellarin treatment of BV‐2 microglial cells resulted in a significant reduction in the protein expression levels and incidence of cells immunopositive for p‐NF‐*κ*B, TNF‐*α*, IL‐1*β*, Bax, and C‐caspase‐3. In contrast, the expression levels of p‐PI3K, p‐AKT, p‐GSK3*β*, and Bcl‐2 were further increased, significantly different from those in the LPS group. The PI3K inhibitor LY294002 had similar effects to scutellarin by inhibiting neuroinflammation and apoptosis in activated microglia. The results of the PI3K/AKT/GSK3*β* signaling pathway and NF‐*κ*B pathway in vivo in MCAO models induced microglia at 3 days were consistent with those obtained from in vitro cells. These findings indicate that scutellarin plays a neuroprotective role by reducing microglial neuroinflammation and apoptosis mediated by the activated PI3K/AKT/GSK3*β*/NF‐*κ*B signaling pathway.

## Graphical abstract

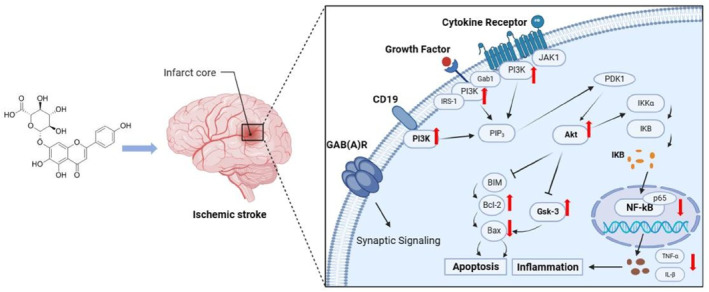

## 1 INTRODUCTION

Ischemic stroke (IS) refers to acute cerebrovascular occlusion in which cerebral ischemia and hypoxia induce localized tissue necrosis.^1^ Although cerebrovascular diseases primarily occur in older people, early onset has been reported at a younger age, which seriously threatens human health and quality of life.^2^ IS is the most prevalent form of cerebrovascular disease, accounting for approximately 60%–80% of stroke cases. It is one of the leading causes of permanent disability and death worldwide.^3^ IS is treated with intravenous tissue plasminogen thrombolysis within 6 h of onset. Owing to the time‐sensitive nature of tissue plasminogen thrombolysis, most patients cannot be treated in time, and up to 3% of patients have fatal or non‐fatal intracranial hemorrhage and other serious complications.^4^, ^5^ Recent comprehensive studies on the pathophysiological mechanism underlying stroke have revealed that oxidative stress, neuroinflammation, calcium overload, oxygen‐free radical formation, and abnormal activation of immune cells play important roles in brain injury after stroke, which eventually leads to neuronal death.^2^, ^6^, ^7^ Although many factors aggravate brain injury, the neuroinflammatory response is the key factor that mediates cascade reactions after IS and is important in its pathogenesis.^8^

Microglia, the first line of immune defense in the central nervous system (CNS), are sensors that monitor and respond to abnormal changes inside and outside the CNS. Under conditions such as IS, microglial activation and migration to the peri‐infarct area aggravates the inflammatory response by producing inflammatory cytokines and cytotoxic substances, affecting adjacent healthy neurons and causing progressive neuronal damage or death.^9^

Plant‐derived natural compounds have gained widespread recognition in recent years owing to their neuroprotective effects against cerebral ischemic injury.^10^ Among them is scutellarin, an effective monomer component of the characteristic Chinese herbal medicine Erigeron scutellarin, which can improve the function of cardiovascular and cerebrovascular diseases. It has a variety of pharmacological activities, including anti‐oxidative, anti‐inflammatory, vascular relaxation, anti‐platelet, anticoagulation, and myocardial protection.^11^ Studies have revealed that scutellarin can reduce oxidative damage and has anti‐apoptotic properties by down‐regulating NOX2 in animal models of IS. Furthermore, it was considered to have potential therapeutic value in treating Alzheimer's disease, cancer, diabetic vascular complications, and other diseases.^10^, ^12^ Our previous studies have demonstrated that scutellarin and edaravone, alone or in combination, can down‐regulate lipopolysaccharide (LPS)‐activated BV‐2 microglia‐mediated inflammation and play a neuroprotective role. However, the molecular targets of scutellarin have not been fully elucidated.

Therefore, the present study used middle cerebral artery occlusion (MCAO) rats and LPS‐induced BV‐2 microglia to simulate the IS model, combined with network pharmacology and experimental validation, to identify a new target pathway of scutellarin for treating IS.

## 2 MATERIALS AND METHODS

### 2.1 Bioinformatics analysis

PharmMapper^13^ and SwissTargetPrediction^14^ databases were used to identify the effective targets of scutellarin. The targets were standardized using the UniProt database.^15^ OMIM,^16^ GeneCards,^17^ and DrugBank^18^ were used to obtain the effective disease targets with the keywords “cerebral ischemia” and “IS”. The disease‐associated drug targets were cross‐matched using a micro‐letter website to build a visualization venny diagram. The intersection targets were then constructed using the STRING^19^ database and protein interaction (PPI) network. Cytoscape 3.7.2^20^ was employed to visualize the PPI network and draw a PPI network diagram. The Metascape database^21^ was deployed to perform gene ontology (GO) enrichment analysis of the intersecting genes, including biological process (BP), cellular component (CC), and molecular function (MF). Kyoto encyclopedia of genes and genomes (KEGG) analysis was performed to identify the signaling pathways with significant enrichment of active components. The results were imported into a micro‐letter website mapping software for visual analysis. AutoDock Vina1.5.6 was used to verify the reliability of the predicted scutellarin and key targets. The structure was dehydrated and hydrogenated using Pymol software, followed by AutoDock Vina1.5.6 docking of the protein receptor and active ligand components. The result with the lowest binding energy was selected as the molecular‐docking result.

### 2.2 Animals and experiments were grouped

A total of X male adult Sprague‐Dawley (SD) rats, clean grade, weighing 250–280 g, were provided by the Laboratory Animal Center of Kunming Medical University. The animals were randomly divided into sham operation + normal saline (sham), MCAO + normal saline (MCAO), and MCAO + scutellarin groups. The time point was 3 days (*n* = 3 in each group). The rats in the MCAO group were anaesthetized by intraperitoneal injection of sodium pentobarbital (50 mg/kg), operated with a dental drill into the right parietal bone, and the trunk of the middle cerebral artery (MCA) was blocked. The same surgical procedure was performed in the sham‐operated rats, but the MCA remained intact.

### 2.3 The rats were intraperitoneally injected with scutellarin

Each drug group was intraperitoneally injected with scutellarin (100 mg/kg) dissolved in normal saline (99% purity, Shanghai Weiwei Medical Science and Technology) 2 h before surgery and 12, 24, 36, 48, and 60 h after surgery. Normal saline was administered to rats in both the sham operation and MCAO groups at the same time, dose, and method as in the administration group. The rats were sacrificed 3 days after surgery, and their tissues were extracted.

### 2.4 Recovery of cerebral blood flow was measured by laser speckle imaging system

The rats in each group were anaesthetized with an intraperitoneal injection of pentobarbital sodium (50 mg/kg). The cranium was exposed through an incision in the parietal skin. RFLSI III blood perfusion imaging system was used to measure cerebral blood flow perfusion, and images were taken and analyzed using RFLSI software.

### 2.5 2, 3, 5‐triphenyl tetrazolium chloride

The brain tissue (coronal section) was obtained immediately after sacrifice on the third day and cut into 2 mm brain slices using a mold. Brain sections were stained with a 2% triphenyl tetrazolium chloride (TTC) solution at 37°C for 30 min. Each section was photographed using a camera and analyzed using Image J software. Infarct size was calculated as a percentage of the total infarct area.^22^

### 2.6 Culture and treatment of BV‐2 microglia

BV‐2 microglia (ATCC) were cultured in dulbecco's modified eagle medium (DMEM) supplemented with 10% fetal bovine serum and 1% double antibody in a sterile 5% carbon dioxide incubator at 37°C. The cells were divided into control group (Con), PI3K inhibitor (LY294002) group (I), LPS induction group (LPS), LPS + LY294002 group (LPS + I), LPS + scutellarin intervention group (LPS + S), and LPS + scutellarin + LY294002 group (LPS + S + I). Cells were seeded in six‐well plates at a density of 6 × 10^5^ cells/well. Cells were pretreated with PI3K inhibitor at a dose of 10 μM for 1 h according to the manufacturer's instructions and washed thoroughly with phosphate‐buffered saline (PBS). Subsequently, the cells were pretreated with scutellarin (0.54 μM) for 1 h at 37°C in a sterile incubator with 5% carbon dioxide (the amount of scutellarin was based on the cell viability assay determined in our previous study^23^). After incubation, the medium was discarded, and the cells were washed with PBS and treated with LPS (1 mg/mL, Sigma‐Aldrich) for 3 h. The medium was replaced with DMEM without serum or double antibody before treatment. For the control group, the medium was replaced with DMEM without serum and double‐antibody.

### 2.7 Tissue and BV‐2 cell Western blotting analysis

Cortical tissue from ischemic areas was stripped and added to lysis buffer (1 × RIPA lysis buffer, protease inhibitor mixture, and phosphatase inhibitor mixture). After centrifugation, the supernatant was removed carefully. The BV‐2 microglia were washed twice with PBS, incubated at 4°C, and lysed with lysis buffer for 15 min. The samples were scraped with a rubber scraper and centrifuged at 14,000 rpm for 15 min. The bicinchoninic acid (BCA) method was used to determine protein concentrations in the tissues and BV‐2 cells. The samples were heated at 95°C for 5 min and separated by electrophoresis on 10% or 12% sodium dodecyl sulfate‐polyacrylamide gels. Protein bands were blotted onto polyvinylidene fluoride (PVDF) membranes and blocked with 5% skim milk powder for 2 h. After washing with tris buffered saline with tween‐20 (TBST), the membranes were treated respectively with PI3K (Mouse Anti‐IgG, 1:1000; Protein‐tech, USA, #60225‐1‐Ig), p‐PI3K (Rabbit Anti‐IgG, 1:1000; Bioss, Beijing, #bs‐5570R), AKT (Rabbit Anti‐IgG, 1:2000; Protein‐tech, USA, #10176‐2‐AP), p‐AKT (Mouse Anti‐IgG, 1:1000; Protein‐tech, USA, #66444‐1‐Ig), GSK3β (Rabbit AntiIgG, 1:1000; Protein‐tech, USA, #22104‐1‐AP), p‐GSK3β (Rabbit Anti‐IgG, 1:1000; Bioss, Beijing, #bs‐2066R), NF‐κB (Rabbit Anti‐IgG, 1:1000; Protein‐tech, USA, #80979‐1‐RR), p‐NF‐*κ*B (Rabbit Anti‐IgG, 1:1000; Bioss, Beijing, #bs‐0982R), TNF‐α (Mouse Anti‐IgG, 1:1000; Protein‐tech, USA, #60291‐1‐Ig), IL‐1β (Rabbit Anti‐IgG, 1:1000; Affinity, Jiangsu, #AF5103), Bax (Mouse Anti‐IgG, 1:1000; Bioss, Beijing, #bs‐0127M), Bcl‐2 (Rabbit Anti‐IgG, 1:1000; Bioss, Beijing, #bs‐0032R), caspase‐3 (Rabbit Anti‐IgG, 1:1000; Protein‐tech, USA, #19677‐1‐AP), and cleaved‐caspase‐3 (Rabbit Anti‐IgG, 1:1000; Affinity, Jiangsu, #AF7022) overnight at 4°C. The next day, they were washed with TBST and incubated with secondary antibodies conjugated with horseradish peroxidase (HRP)‐conjugated anti‐rabbit IgG (dilution 1:500; Chemicon, #AP32P) or anti‐mouse IgG (dilution 1:500; Chemicon, #AP32P) separately. The membranes were horizontally cut into multiple pieces according to their molecular weights to probe different proteins. The enhanced chemiluminescence (ECL) reagent was used to develop the gel imaging system and label the results. The Image J software was used to analyze the experimental data.

### 2.8 Double immunofluorescence staining of tissues and BV‐2 cells was performed

Rats were sacrificed 3 days after being anaesthetized with 3% sodium pentobarbital. The brains were removed and embedded in an optimal cutting temperature compound embedding medium. Coronal sections of the brain were cut to a thickness of 10 μm using a microcutter. The sections were then washed with PBS. To block the nonspecific binding of proteins, tissue sections were incubated in goat serum diluted in 5% PBS for 1 h at room temperature. BV‐2 cells were fixed with 4% paraformaldehyde in 0.1 M PBS for 20 min. After washing with PBS, the plates were incubated with goat serum diluted in 5% PBS for 1 h at room temperature. In each group, tissue sections or BV‐2 cells were incubated with the primary antibody (1:200) overnight at 4°C. Subsequently, sections were incubated with Lectin (1:200, SIGMA, #SLPB1894V)/Cy3 (1:200, Jackson Immuno‐Research, #125364) in the dark at room temperature for 1 h. After washing, coverslips were sealed with a fluorescent mounting agent containing 4′, 6‐diamidino‐2‐phenylindole (DAPI). Colocalization was observed using immunofluorescence microscopy (Zeiss cell observer, Zeiss CaT #1026470987). Image J software was used for quantification. The intensity changes were then plotted.

### 2.9 Statistics analyses

Data were expressed as the mean ± standard deviation (X ± SD). Statistical significance was assessed using a one‐way analysis of variance. Differences were considered statistically significant at *p* < 0.05. GraphPad Prism software (version 8.0) was used to analyze all data.

## 3 RESULTS

### 3.1 Network pharmacology and molecular docking

**FIGURE 1:**
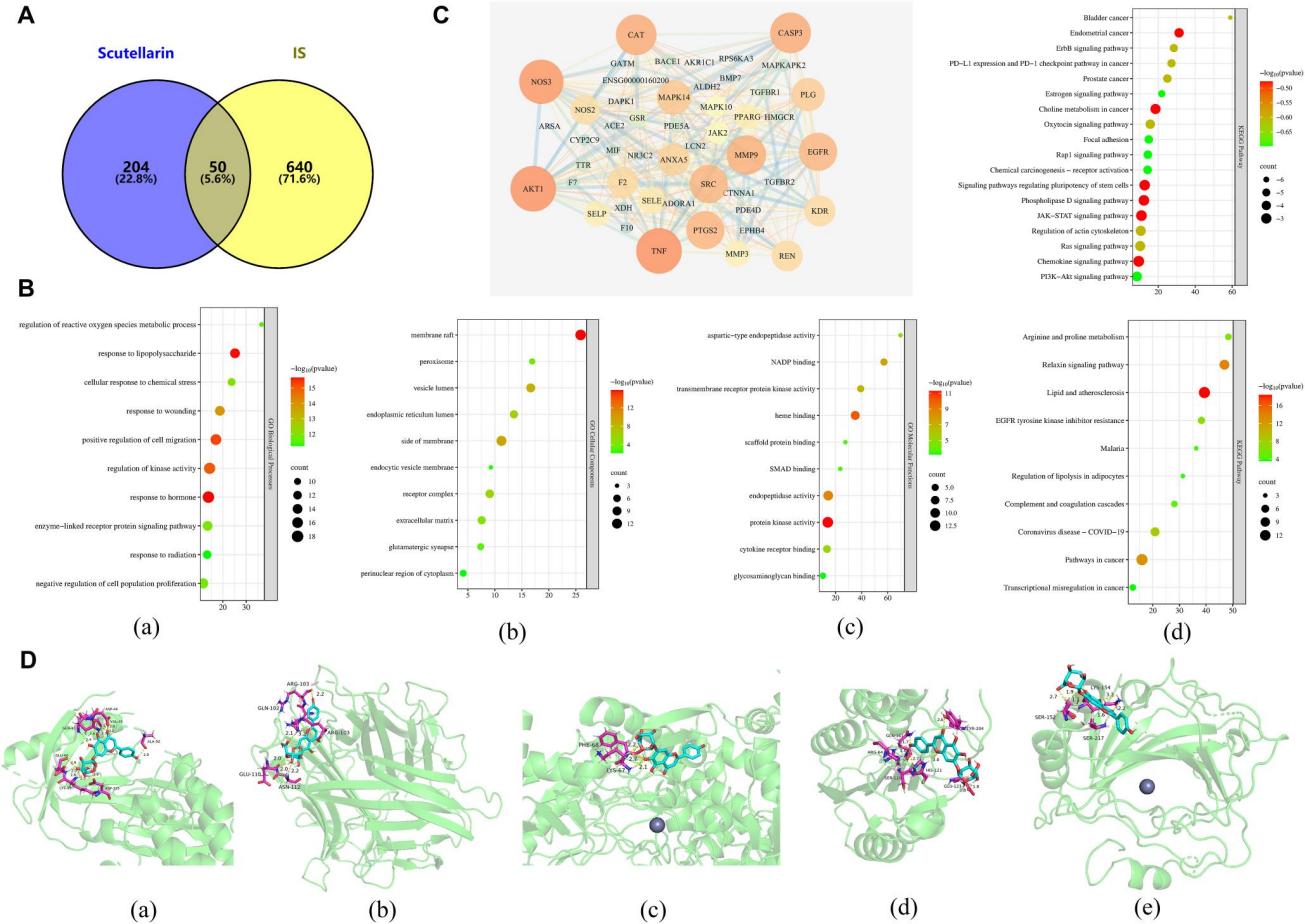
Network pharmacology and molecular docking. (A) Venn diagram of the intersection targets. (B) GO function and KEGG pathway enrichment analysis; (a) biological processes; (b) cellular components; (c) molecular functions; (d) KEGG. (C) PPI network and KEGG pathway analyses. (D) Visualization of molecular docking between scutellarin and core targets; (a) Scutellarin and AKT1, (b) Scutellarin and TNF; (c) Scutellarin and NOS3; (d) Scutellarin and CASP3; (e) Scutellarin and CAT. GO, gene ontology; KEGG, kyoto encyclopedia of genes and genomes.

Based on PharmMapper and SwissTargetPrediction prediction results, 254 targets were predicted for scutellarin, and duplicated targets were removed. A total of 690 potential IS targets were obtained from the OMIM, GeneCards, and DrugBank databases. Venny diagram was constructed to obtain 50 intersection targets (Figure 1A). Functional enrichment analysis was performed on the intersection targets. GO and KEGG functional enrichment analyses from the Metascape database revealed that BP mainly focused on the positive regulation of hormones, response to LPS, and positive regulation of cell migration; CC on the side of the membrane, vesicle lumen, and receptor complexes; and MF on protein kinase activity, heme binding, and endopeptidase activity. KEGG pathways were mainly involved in lipid and atherosclerosis, the relaxin signaling pathway, and pathways in cancer (Figure 1B). PPI networks were constructed by importing intersection targets into the STRING database and visualizing them using Cytoscape 3.7.2. The network consisted of 50 targets and 285 edges. Larger nodes indicate a higher importance. According to the degree value, the top five core proteins were selected for molecular docking, including AKT1 (Degree 30), TNF (Degree 30), NOS3 (Degree 29), CASP3 (Degree 26), and CAT (Degree 26).

NOS3 is the most important isoform of NO produced in the cardiovascular system.^24^ CAT plays an important role in protecting cells against hydrogen peroxide toxicity.^25^ Notably, activation of AKT1, an isoform of AKT, promotes cell survival in multiple ways and is closely related to neuronal survival in ischemic diseases of the CNS.^26^, ^27^ Further screening of AKT1 using enriched signaling pathways revealed that the PI3K/AKT signaling pathway is crucial in regulating inflammation and apoptosis. Therefore, the PI3K/AKT signaling pathway was selected for subsequent experimental validation (Figure 1C). Molecular docking results demonstrated that scutellarin has a certain binding ability with the top five core target proteins. Binding energy, AKT1 (−5.19 kcal/mol), TNF (−6.31 kcal/mol), NOS3 (−3.84 kcal/mol), CASP3 (−4.15 kcal/mol), and CAT (−5.86 kcal/mol) (Figure 1D).

### 3.2 Effect of scutellarin on PI3K/AKT/GSK3*β* signaling pathway and NF‐*κ*B expression in BV‐2 microglia cells activated by LPS

**FIGURE 2:**
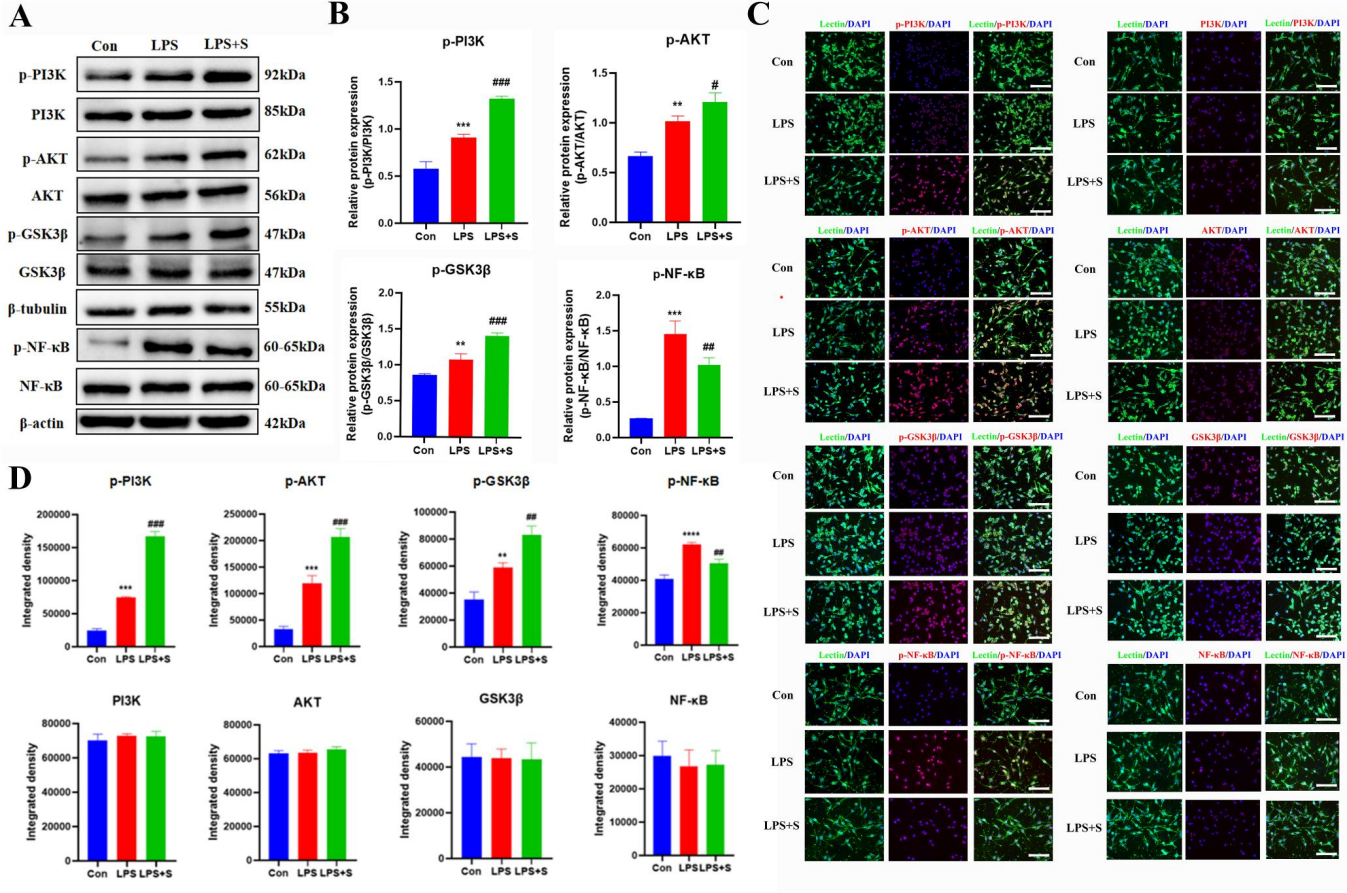
Effect of scutellarin on the expression and fluorescence intensity of related proteins in LPS‐induced BV‐2 microglial cells. (A, B) Western blotting bands and quantitative statistical plots. (C, D) Immunofluorescence images and quantitative statistics. The target factor (red), lectin (green), is a marker for microglia, and DAPI (blue) shows the nuclei. *p < 0.05, **p < 0.01, ***p < 0.001, ****p < 0.001 between control and LPS groups; #*p* < 0.05, ##*p* < 0.01, ###*p* < 0.001 between LPS and LPS + S groups. Scale bar = 50 μm. The values are represented as the mean ± SD of triplicate experiments.

Western blotting analysis revealed a statistically significant increase in the protein expression of p‐PI3K, p‐AKT, p‐GSK3*β*, and p‐NF‐*κ*B in the LPS group compared to the control group. In addition, the scutellarin pretreatment group had an even higher expression of p‐PI3K, p‐AKT, and p‐GSK3*β* proteins, whereas p‐NF‐*κ*B protein expression was significantly decreased (Figure 2A,B).

Immunofluorescence analysis indicated that the incidence of immunofluorescence p‐PI3K, p‐AKT, p‐GSK3*β*, and p‐NF‐*κ*B positive cells was higher in the LPS group than in the control group. Scutellarin increased the fluorescence intensity of p‐PI3K, p‐AKT, and p‐GSK3*β*, whereas the fluorescence intensity of p‐NF‐*κ*B significantly decreased. The results of PI3K, AKT, GSK3*β*, and NF‐*κ*B were unchanged among the groups (Figure 2C,D).

### 3.3 Effect of scutellarin on the expression of TNF‐*α* and IL‐1*β* in BV‐2 microglial cells activated by LPS

**FIGURE 3:**
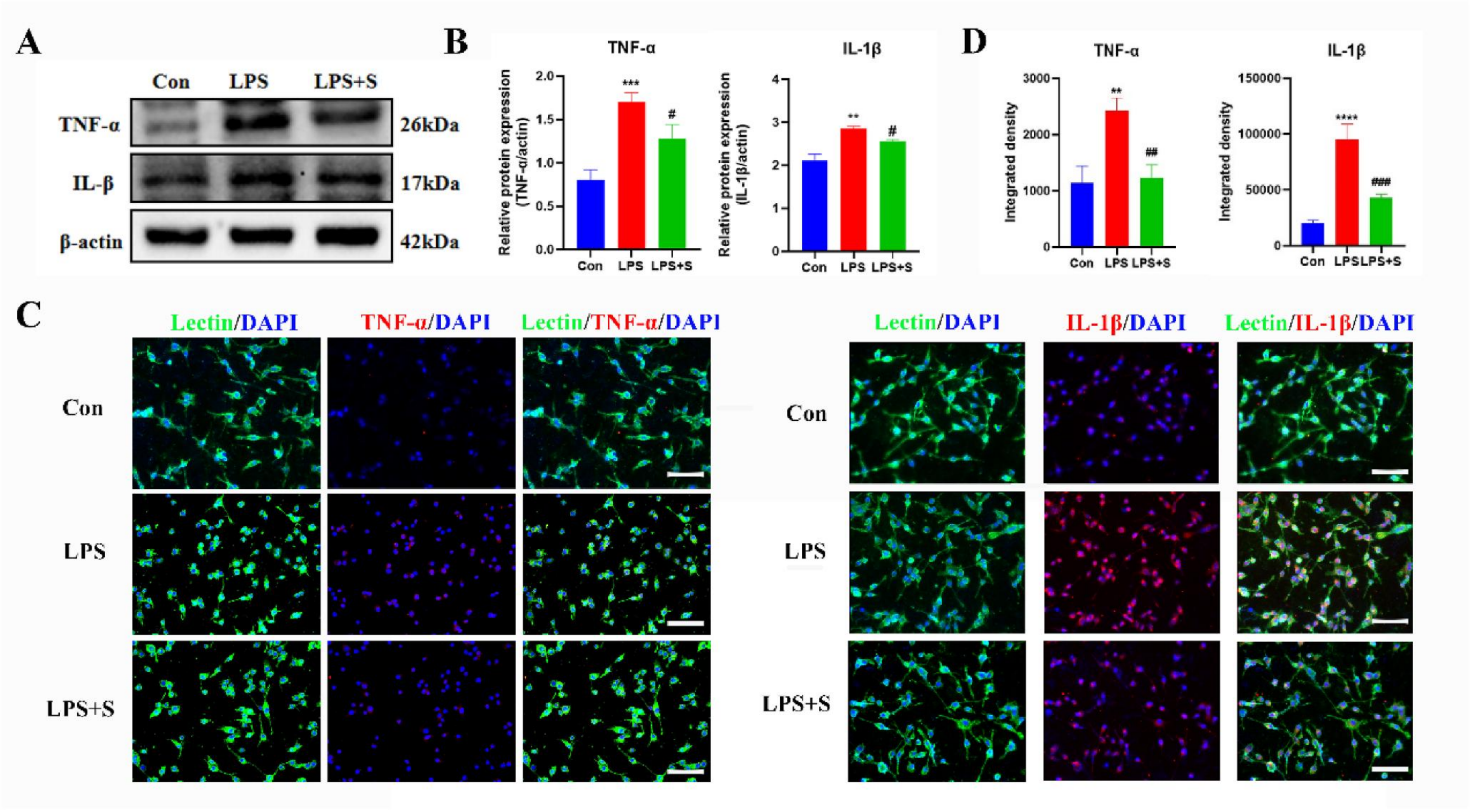
Effect of scutellarin on protein expression and fluorescence intensity of inflammatory factors in LPS‐stimulated BV‐2 microglial cells. (A, B) Western blotting bands and quantitative statistical plots. (C, D) Immunofluorescence images and quantitative statistics. The target factor (red), lectin (green), is a marker for microglia, and DAPI (blue) shows the nuclei. **p < 0.01, ***p < 0.001, ****p < 0.001 between control and LPS groups; #*p* < 0.05, ##*p* < 0.01, ###*p* < 0.001 between LPS and LPS + S groups. Scale bar = 50 μm. Values represent the mean ± SD of triplicate experiments.

Western blotting analysis revealed a statistically significant increase in the protein expression of TNF‐α and IL‐1β in the LPS group compared to the control group, whereas it was significantly decreased by scutellarin treatment (Figure 3A,B). The immunofluorescence results were consistent with those of western blotting (Figure 3C,D).

### 3.4 Effect of scutellarin on the expression of apoptotic proteins in BV‐2 microglia cells activated by LPS

**FIGURE 4:**
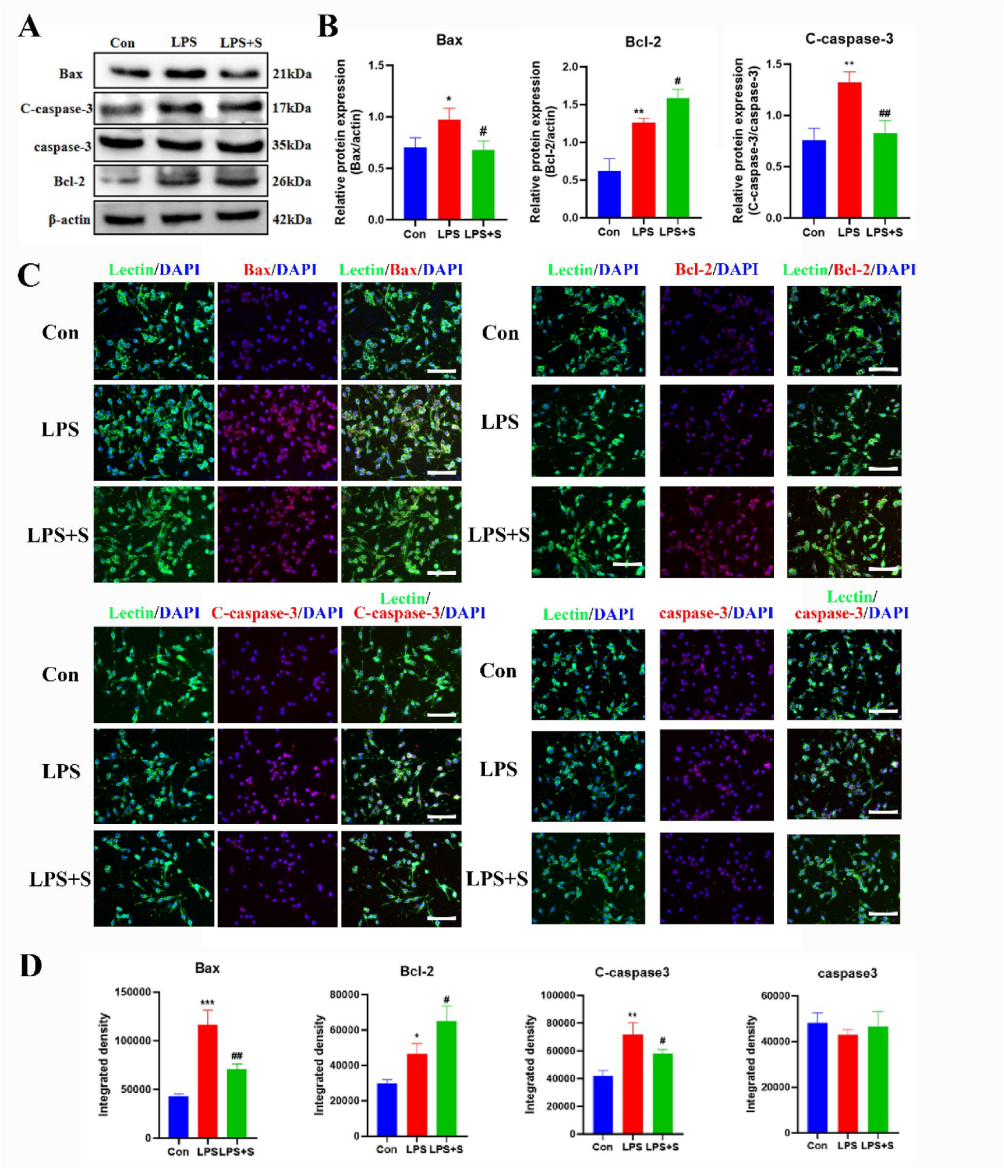
Effect of scutellarin on protein expression and fluorescence intensity of apoptosis in LPS‐induced BV‐2 microglial cells. (A, B) Western bolt bands and quantitative statistical plots. (C, D) Immunofluorescence images and quantitative statistics. The target factor (red), lectin (green), is a marker for microglia, and DAPI (blue) shows nuclei. *p < 0.05, **p < 0.01, ***p < 0.001 between control and LPS groups; #*p* < 0.05, ##*p* < 0.01 between LPS and LPS + S groups. Scale bar = 50 μm. Values represent the mean ± SD of triplicate experiments.

Western blotting analysis revealed a statistically significant increase in the protein expression of cleaved caspase‐3, Bax, and Bcl‐2 in the LPS group compared to that in the control group. After scutellarin treatment, the expression of cleaved caspase‐3 and Bax proteins significantly decreased. In contrast, the Bcl‐2 expression, an anti‐apoptotic protein, further increased (Figure 4A,B). The immunofluorescence results were consistent with the Western blotting results. There was no significant difference in the expression of caspase‐3 between the groups (Figure 4C,D).

### 3.5 Effects of PI3K pathway inhibitor LY294002 on the expression of PI3K/AKT/GSK3*β* and NF‐*κ*B pathway in LPS‐activated BV‐2 microglial cells

**FIGURE 5:**
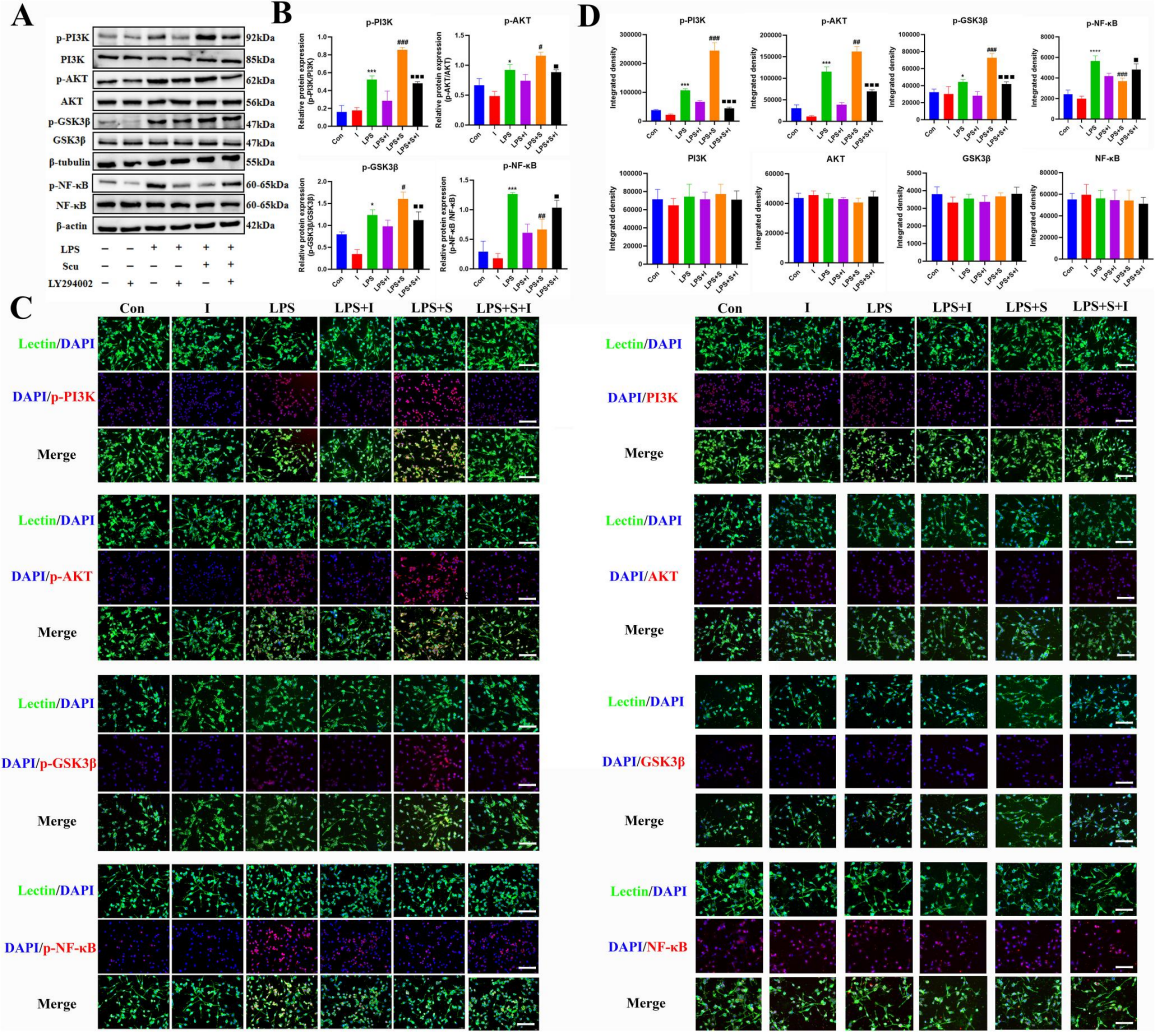
Effects of LY294002 on the expression and fluorescence intensity of pathway‐related proteins in LPS‐stimulated BV‐2 microglial cells. (A, B) Western blotting bands and quantitative statistical plots. (C, D) Immunofluorescence images and quantitative statistics. The target factor (red), lectin (green), is a marker for microglia, and DAPI (blue) shows the nuclei. *p < 0.05, ***p < 0.001, ****p < 0.001 between control and LPS group; #*p* < 0.05, ##*p* < 0.01, ###*p* < 0.001 between LPS and LPS + S groups; ■*p* < 0.05, ■■*p* < 0.01, ■■■*p* < 0.001 between LPS + S and LPS + S + I groups. Scale bar = 50 μm. Values represent the mean ± SD of triplicate experiments.

The results of Western blotting (Figure 5A,B) and immunofluorescence (Figure 5C,D) revealed that the administration of scutellarin resulted in a significant increase in the expression of p‐PI3K, p‐AKT, and p‐GSK3*β* related proteins, whereas the expression of p‐NF‐*κ*B protein was significantly decreased compared to that in the LPS group. After treatment with LY294002 (LPS + S + I), the protein expression of p‐PI3K, p‐AKT, and p‐GSK3*β* significantly decreased, whereas the protein expression of p‐NF‐*κ*B significantly increased. The difference was statistically significant compared to the scutellarin intervention group (LPS + S). However, the fluorescence intensities of PI3K, AKT, GSK3*β*, and NF‐*κ*B did not significantly differ between the groups.

### 3.6 Effects of PI3K pathway inhibitor LY294002 on the expression of inflammatory factors TNF‐*α* and IL‐1*β* in BV‐2 microglia cells activated by LPS

**FIGURE 6:**
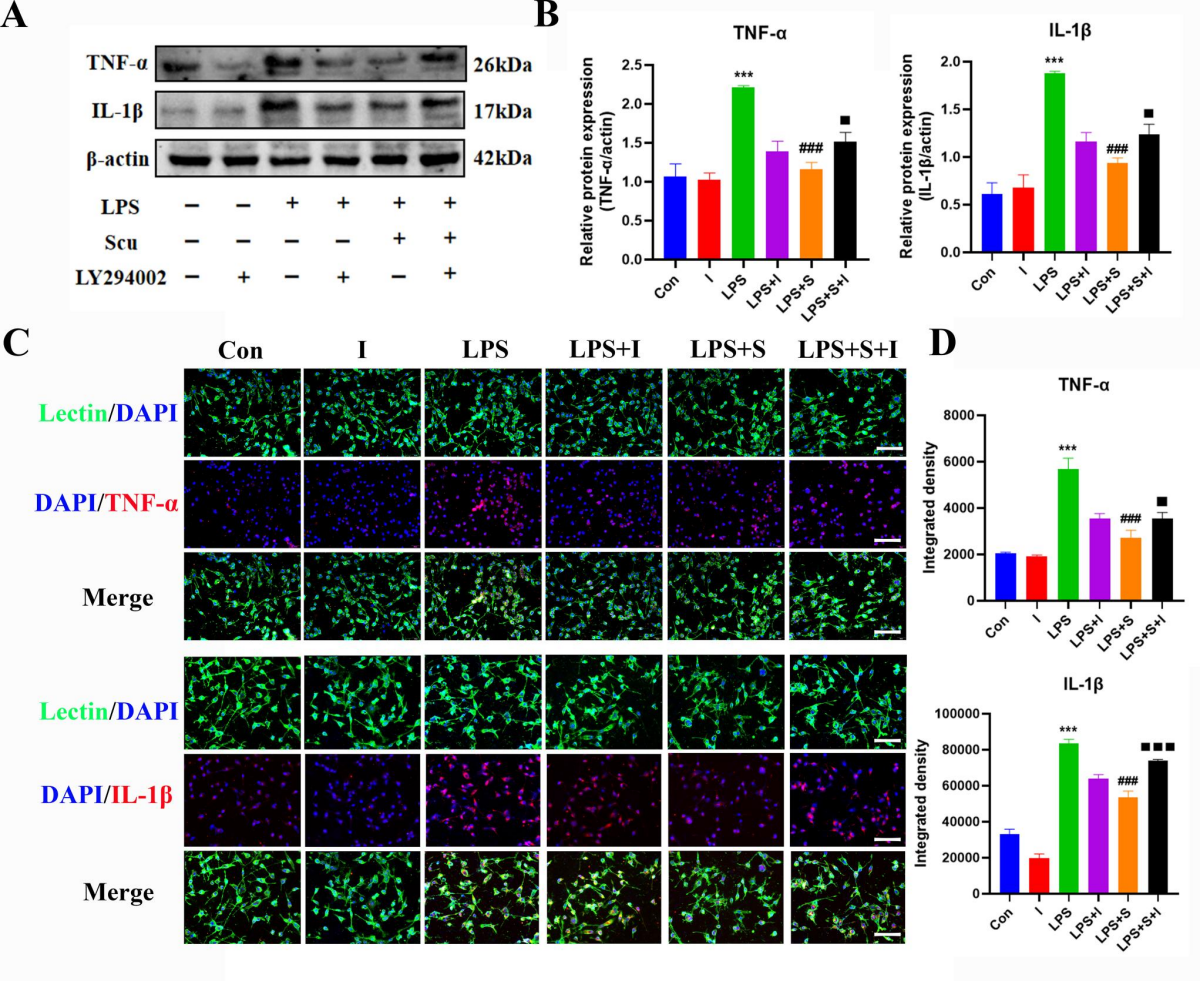
Effects of LY294002 on protein expression and fluorescence intensity of inflammatory factors in LPS‐induced BV‐2 microglial cells. (A, B) Western bolt bands and quantitative statistical plots. (C, D) Immunofluorescence images and quantitative statistics. The target factor (red), lectin (green), is a marker for microglia, and DAPI (blue) shows the nuclei. ***p < 0.001 between control and LPS groups; ###*p* < 0.001 between LPS and LPS + S groups; ■*p* < 0.05, ■■■*p* < 0.001 between LPS + S and LPS + S + I groups. Scale bar = 50 μm. Values represent the mean ± SD of triplicate experiments.

The results of western blotting (Figure 6A,B) and immunofluorescence (Figure 6C,D) analyses revealed that the expression of TNF‐α and IL‐1β proteins in the scutellarin intervention group was significantly decreased compared with that in the LPS group. After treatment with LY294002 (LPS + S + I), TNF‐α and IL‐1β protein expression increased, compared with the scutellarin intervention group (LPS + S).

### 3.7 Effect of PI3K pathway inhibitor LY294002 on the expression of apoptotic proteins in BV‐2 microglia cells activated by LPS

**FIGURE 7:**
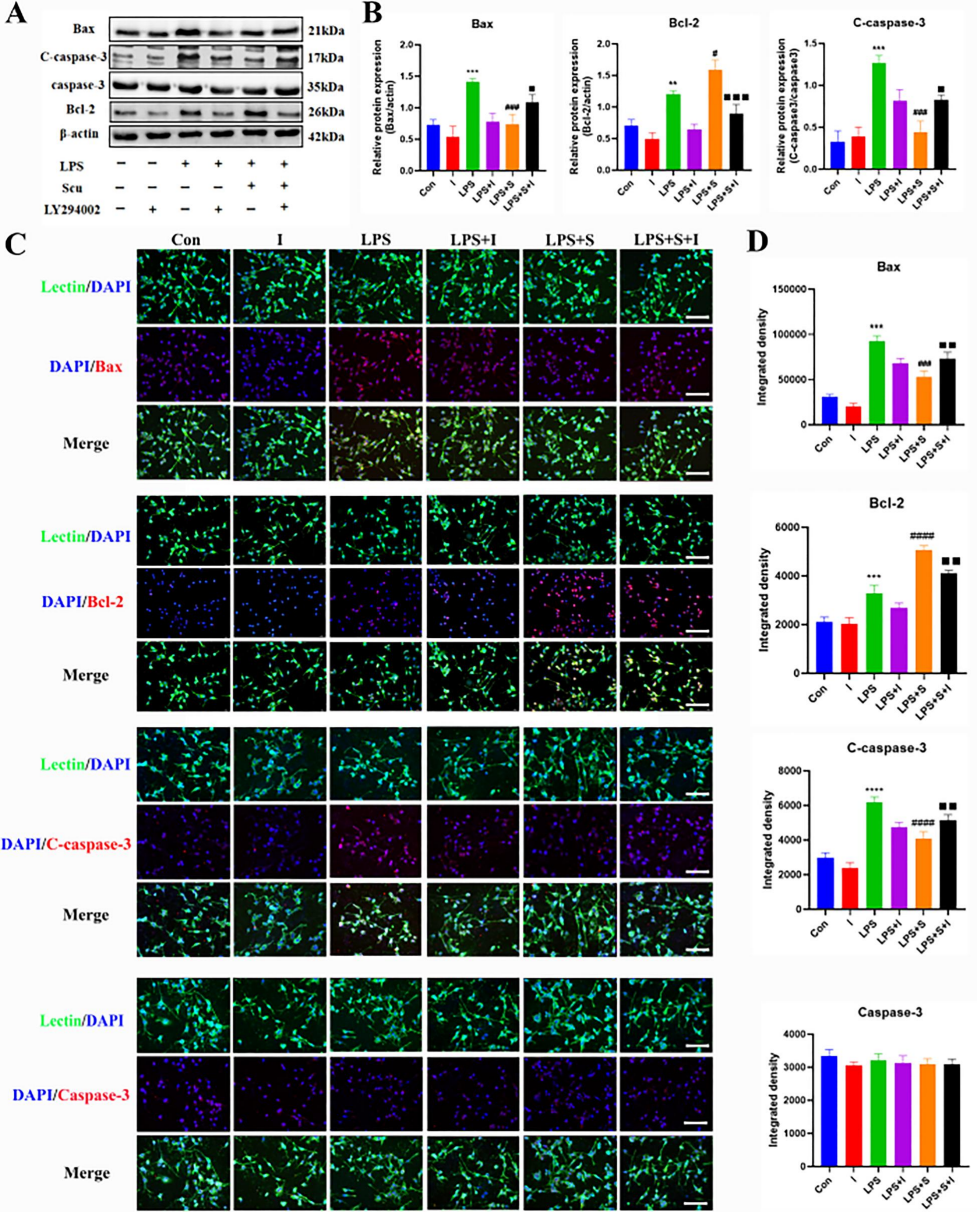
Effects of LY294002 on protein expression and fluorescence intensity of apoptotic factors in BV‐2 microglial cells induced by LPS. (A, B) Western bolt bands and quantitative statistical plots. (C, D) Immunofluorescence images and quantitative statistics. The target factor (red), lectin (green), is a marker for microglia, and DAPI (blue) shows the nuclei. **p < 0.01, ***p < 0.001, ****p < 0.001 between control and LPS groups; #*p* < 0.05, ###*p* < 0.001, ####*p* < 0.001 between LPS and LPS + S groups; ■*p* < 0.05, ■■*p* < 0.01, ■■■*p* < 0.001 between LPS + S and LPS + S + I groups. Scale bar = 50 μm. Values represent the mean ± SD of triplicate experiments.

The results of Western blotting (Figure 7A,B) and immunofluorescence (Figure 7C,D) analyses demonstrated that the expression of cleaved caspase‐3 and Bax proteins was decreased, whereas the expression of Bcl‐2 protein was further increased in the scutellarin pretreatment group compared to the LPS group. LY294002 (LPS + S + I) treatment up‐regulated cleaved caspase‐3 and Bax protein expression, whereas Bcl‐2 protein expression was down‐regulated. There was no significant difference in the fluorescence intensity of caspase‐3 between the groups.

### 3.8 Cerebral blood flow imaging and TTC staining in 3‐day MCAO rats

**FIGURE 8:**
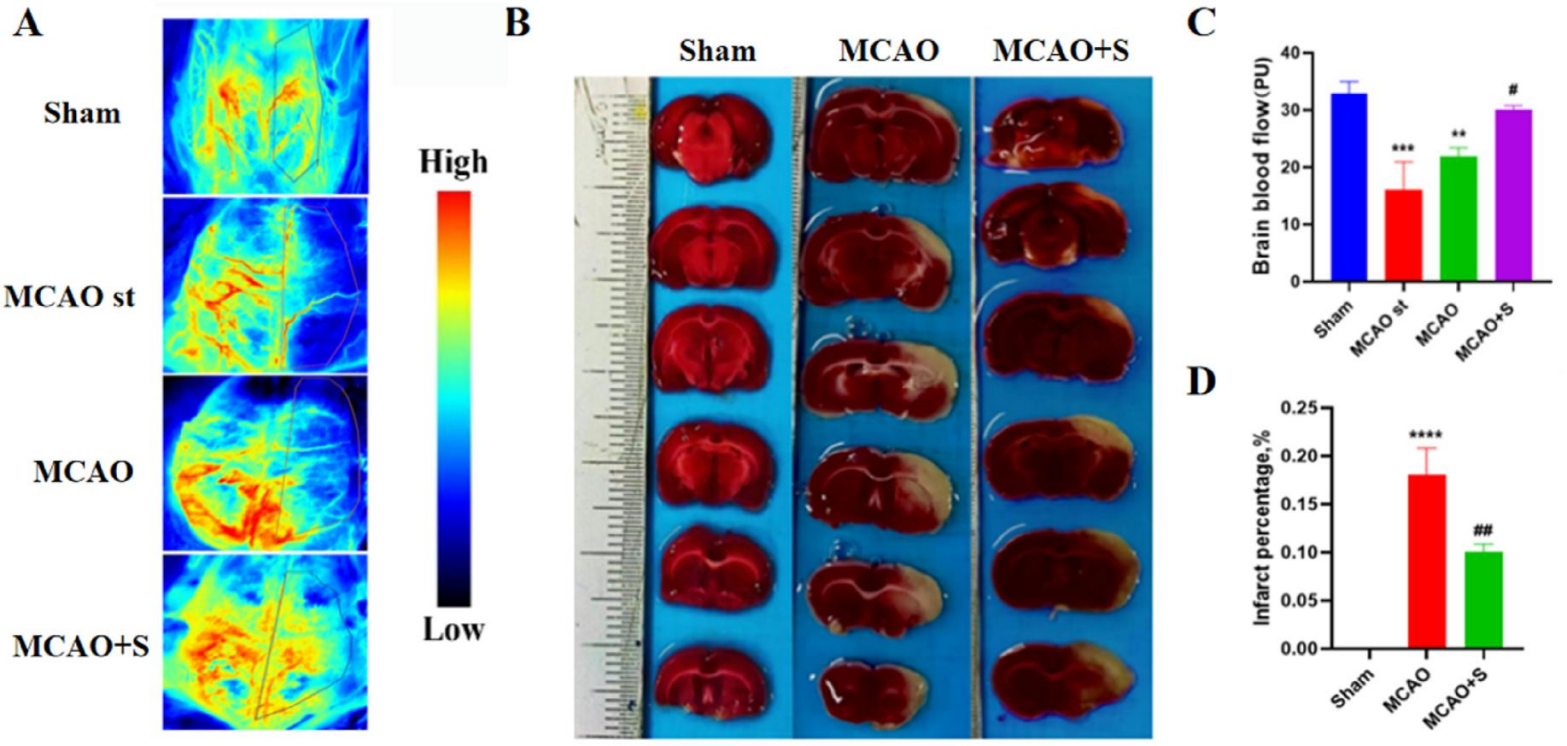
Cerebral blood flow imaging and TTC staining in 3‐day MCAO rats. (A) Laser speckle imaging was used to observe the recovery of cerebral blood flow in MCAO rats for 3 days. (B) TTC staining of rat brain tissue after 3 days. (C) Statistical map of cerebral blood flow recovery. (D) Statistical plot of infarct volume changes. ***p < 0.001, ****p < 0.001 versus sham group; **p < 0.01 versus MCAO‐st group; #*p* < 0.05, ##*p* < 0.01 versus MCAO group.

The results of laser speckle imaging illustrated that rat blood flow was significantly reduced in the MCAO‐st group compared with that in the sham group. Compared with the MCAO‐st group, the MCAO group's blood flow was restored to some extent, but the blood perfusion was not completely restored owing to the establishment of collateral circulation. Compared with the MCAO group, blood flow was significantly improved after the intervention with scutellarin (MCAO + S) (Figure 8A,C).

TTC results revealed that the infarct volume of the brain tissue in the MCAO group was significantly higher than in the sham group. However, it was significantly reduced by pretreatment with scutellarin (Figure 8B,D).

### 3.9 Effect of scutellarin on the expression of PI3K/AKT/GSK3β and NF‐*κ*B pathway in activated microglia in 3‐day MCAO rats

**FIGURE 9:**
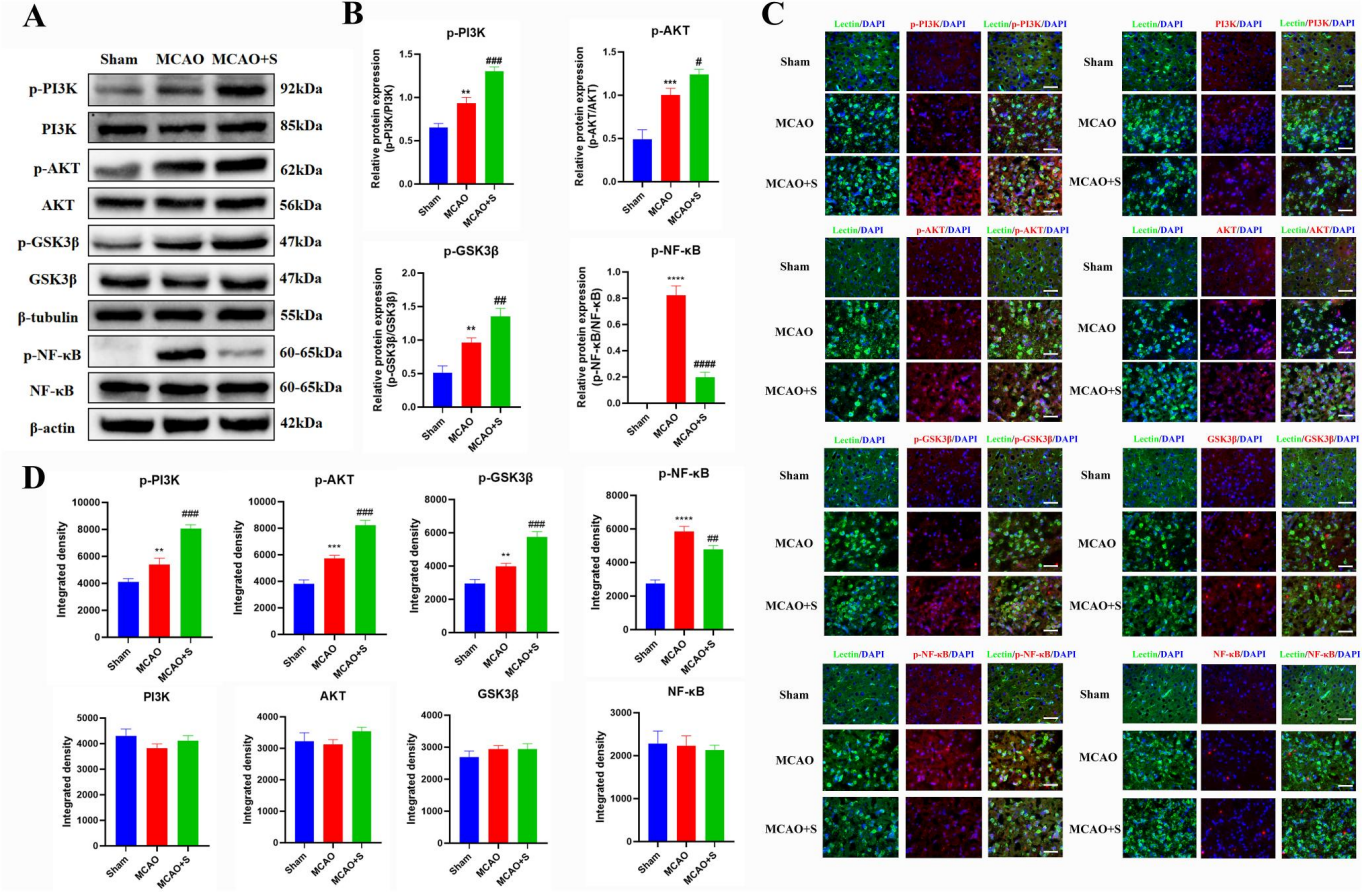
Effect of scutellarin on the expression and fluorescence intensity of pathway‐related proteins in microglia of 3‐day MCAO rats. (A, B) Western bolt bands and quantitative statistical plots. (C, D) Immunofluorescence images and quantitative statistics. The target factor (red), lectin (green), is a marker for microglia, and DAPI (blue) shows the nuclei. **p < 0.01, ***p < 0.001, ****p < 0.001 between sham and MCAO groups; #*p* < 0.05, ##*p* < 0.01, ###*p* < 0.001, ####*p* < 0.001 between MCAO and MCAO + S groups. Scale bar = 25 μm. Values represent the mean ± SD of triplicate experiments.

Western blotting analysis revealed a significant increase in the protein expression of p‐PI3K, p‐AKT, p‐GSK3*β*, and pNF‐κB in the MCAO group on day three compared to the sham operation group. After treatment with scutellarin, the expression of p‐PI3K, p‐AKT, and p‐GSK3*β* related proteins was further increased, whereas the expression of p‐NF‐*κ*B protein was significantly reduced (Figure 9A,B). Double‐labeled fluorescent staining on the cortex of 3 days MCAO rats demonstrated that the fluorescence intensity of p‐PI3K, p‐AKT, p‐GSK3β, and p‐NF‐κB in the MCAO group and the number of positive cells significantly increased. However, scutellarin treatment further increased the fluorescence intensities of p‐PI3K, p‐AKT, and p‐GSK3β while reducing the fluorescence intensity of p‐NF‐κB, and the difference was statistically significant. There was no significant difference in the fluorescence intensity of PI3K, AKT, GSK3β, and NF‐κB between the groups (Figure 9C,D).

### 3.10 Effect of scutellarin on the expression of inflammatory factors TNF‐α and IL‐1β in activated microglia in 3‐day MCAO rats

**FIGURE 10:**
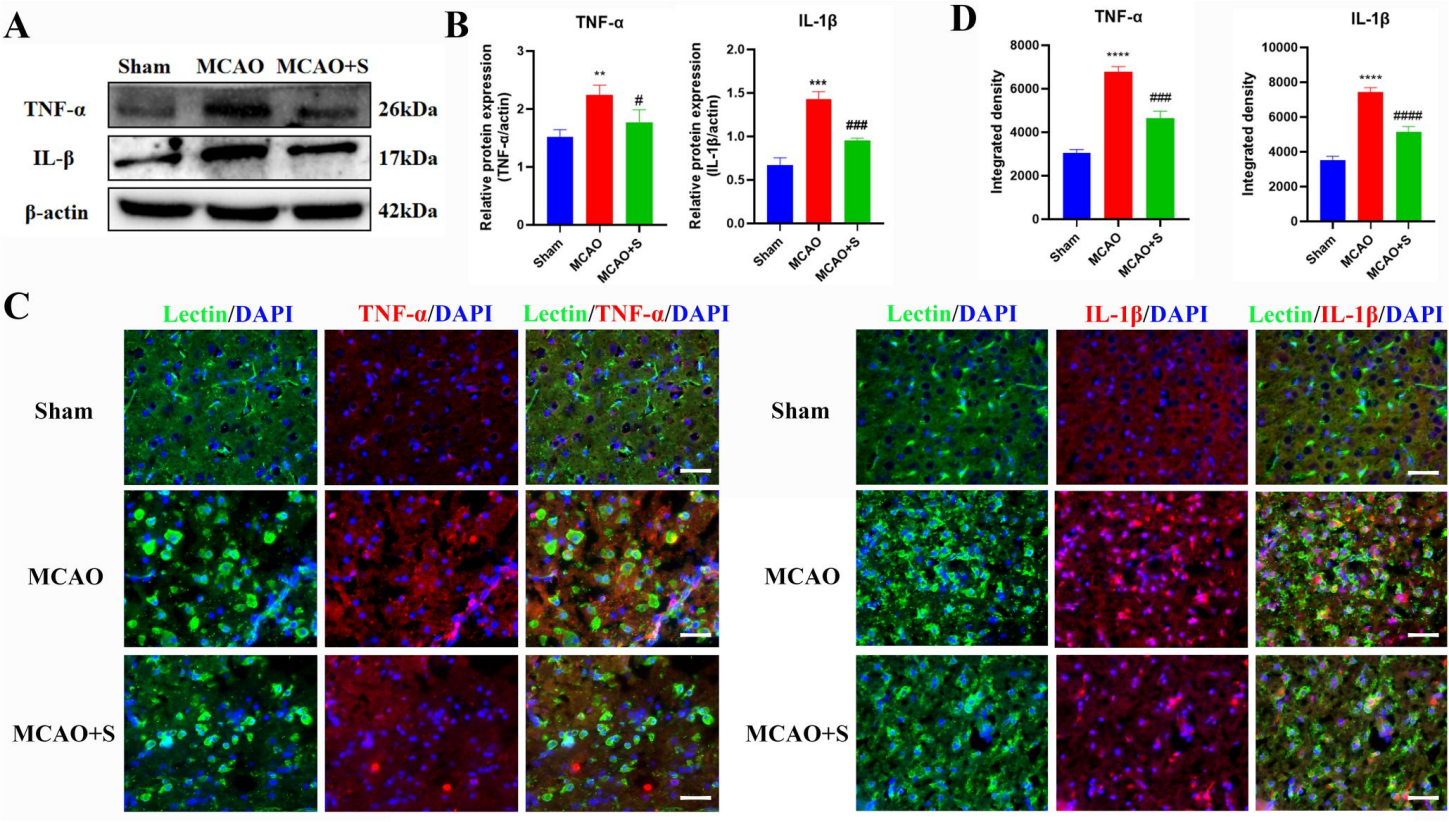
Effect of scutellarin on protein expression and fluorescence intensity of inflammatory factors in microglia of 3‐day MCAO rats. (A, B) Western blot bands and quantitative statistical plots. (C, D) Immunofluorescence images and quantitative statistics. The target factor (red), lectin (green), is a marker for microglia, and DAPI (blue) shows the nuclei. **p < 0.01, ***p < 0.001, ****p < 0.001 between the sham and MCAO group; #*p* < 0.05, ###*p* < 0.001, ####*p* < 0.001 between MCAO and MCAO + S groups. Scale bar = 25 μm. Values represent the mean ± SD of triplicate experiments.

Western blotting analysis revealed a significant increase in the protein expression of TNF‐*α* and IL‐1*β* in the MCAO group at 3 days compared to that in the sham operation group. After scutellarin treatment, the expression levels of TNF‐*α* and IL‐1*β* decreased significantly (Figure 10A,B). The immunofluorescence results were consistent with the western blot results (Figure 10C,D).

### 3.11 Effect of scutellarin on the expression of apoptotic proteins in activated microglia in 3‐day MCAO rats

**FIGURE 11:**
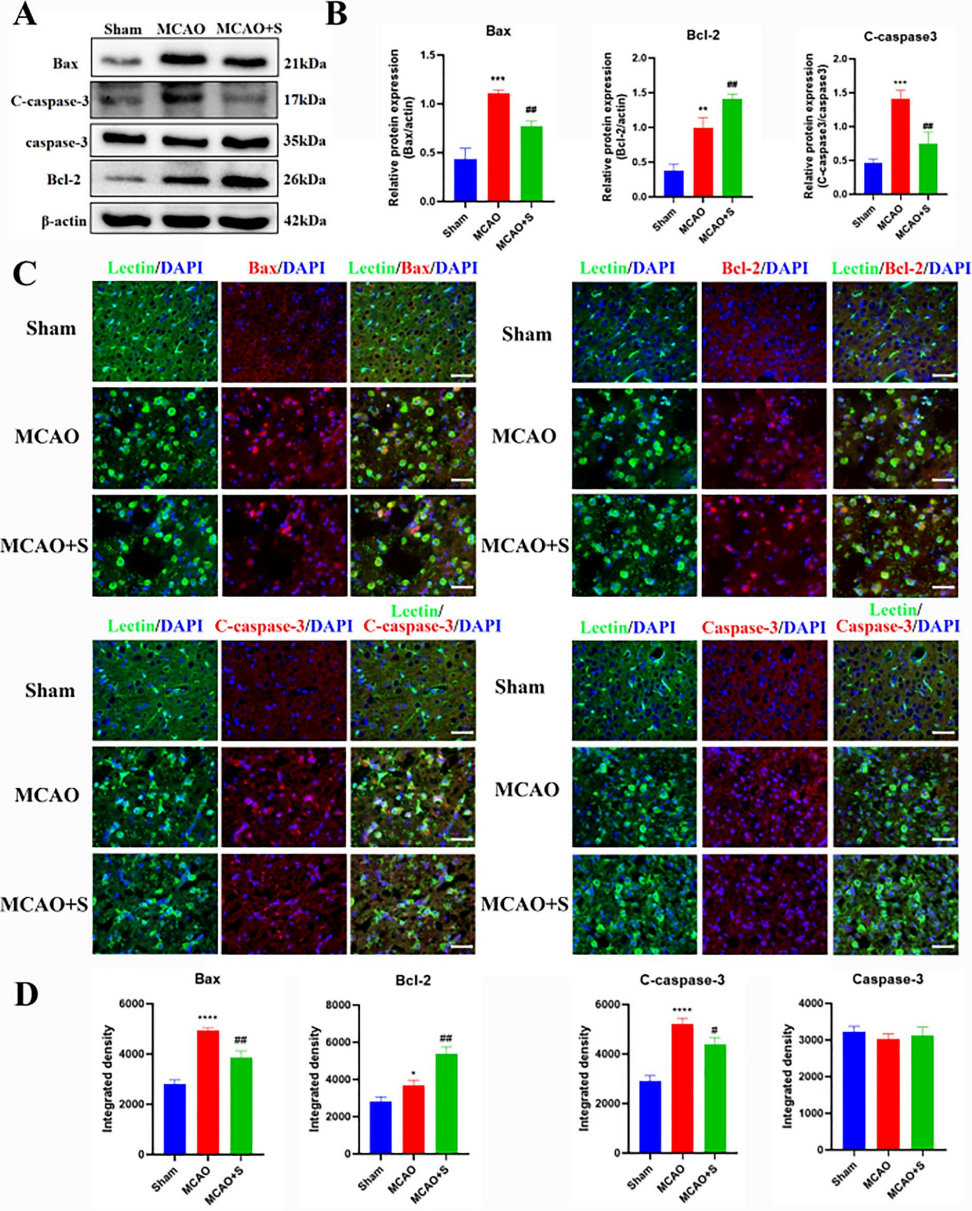
Effect of scutellarin on protein expression and fluorescence intensity of microglial apoptotic factors in 3‐day MCAO rats. (A, B) Western blotting bands and quantitative statistical plots. (C, D) Immunofluorescence images and quantitative statistics. The target factor (red), lectin (green), is a marker for microglia, and DAPI (blue) shows the nuclei. **p < 0.01, ***p < 0.001, ****p < 0.001 between sham and MCAO groups; #*p* < 0.05, ##*p* < 0.01 between MCAO and MCAO + S groups. Scale bar = 25 μm. Values represent the mean ± SD of triplicate experiments.

Western blotting analysis demonstrated a significantly higher expression of pro‐apoptotic proteins cleaved caspase‐3 and Bax and anti‐apoptotic protein Bcl‐2 in the MCAO group than in the sham operation group. After scutellarin treatment, cleaved caspase‐3 and Bax protein expression was significantly reduced. However, the expression of the anti‐apoptotic protein Bcl‐2 was increased further compared to that in the MCAO group (Figure 11A,B). The immunofluorescence results were consistent with the western blot results, except for the expression of caspase‐3 (Figure 11C,D).

## 4 DISCUSSION

Stroke is one of the most serious diseases that causes death and disability in the world. Ischemia and hypoxia of the local brain tissue are caused by cerebral artery occlusion or thromboembolism, leading to inflammatory reactions, oxidative stress, apoptosis, and other malignant cascade reactions. These are serious threats to human life and health.^28^ Microglia are resident immune cells of the CNS that respond in a timely manner to the pathological processes of the CNS, including infection, brain injury, and aging. Microglia primarily respond to neuroinflammation and promote apoptosis.^29^ Natural herbal compounds with neuroprotective effects have recently been widely used to treat CNS‐related diseases. For example, gastrodine promotes activation of the Wnt/*β* Catenin signaling pathway, thereby ameliorating ischemic damage and increasing neurogenesis in mice with focal cerebral ischemia.^5^ Panax notoginsenosides can inhibit microglial activation, reduce the expression of PKM2, and down‐regulate the HIF‐1*α*/PKM2/STAT3 signaling pathway. It has an anti‐inflammatory and neuroprotective role in improving neurofunction.^30^

*Erigeron breviscapus* is a distinct Chinese herb in the Yunnan Province, and scutellarin is an effective monomer component of *Erigeron breviscapus*. Previous studies by our group have demonstrated that scutellarin can promote M2 polarization of microglia by inhibiting the JNK and p38 MAPK signaling pathways while improving the ERK1/2 signaling pathway. Thus, scutellarin exerts anti‐inflammatory and neuroprotective effects.^31^ Studies have also shown that scutellarin combined with edaravone can significantly enhance neurological function and reduce neuronal apoptosis in rats with cerebral ischemia. Various complex signaling molecules and signaling pathways are involved in IS. Therefore, further exploring the related target mechanisms is necessary to provide an experimental basis for the clinical treatment of neurodegenerative diseases such as IS.

The present study used online network pharmacology and molecular docking to explore target proteins related to IS and scutellarin. The analysis revealed that AKT1, TNF, and NOS3 were core target proteins. Scutellarin showed good binding ability with the core target proteins through molecular docking. Further analysis of the signaling pathway enriched by AKT1 revealed that the PI3K/AKT signaling pathway was significantly enriched. AKT1, an AKT family isoform, is involved in the pathogenesis of various diseases.

Phosphatidylinositol 3‐kinase/protein kinase B/glycogen synthase kinase‐3*β* (PI3K/AKT/GSK3β) is an important pathway for maintaining the survival of neurons and cells.^32^ Glycogen synthase kinase‐3*β* (GSK‐3*β*) is an important downstream target of PI3K/AKT and participates in various biological functions, such as neuroinflammation and apoptosis. Activation of GSK‐3*β* can induce NF‐*κ*B and NO production in microglia to produce pro‐inflammatory cytokines. These processes promote microglia migration and mediate neuroinflammation development^33^, ^34^ Studies have reported that oxymatrine plays a neuroprotective role in neonatal hypoxic‐ischemic brain damage by regulating the activation of the PI3K/AKT/GSK3*β* signaling pathway and apoptotic proteins.^35^ The middle cerebral artery occlusion and reperfusion (MCAO/R) model has demonstrated that activation of the PI3K/AKT signaling pathway inhibits inflammation, oxidative stress, and apoptosis, increases BV‐2 cell activity, and promotes PI3K phosphorylation, thus improving the symptoms of neurological dysfunction, such as cerebral ischemia and reperfusion.^36^

Using laser speckle imaging and TTC, our study demonstrated that scutellarin significantly promoted the recovery of cerebral blood flow and reduced cerebral infarction volume in MCAO rats. The expression of p‐PI3K, p‐AKT, p‐GSK3*β*, p‐NF‐*κ*B, inflammatory factors TNF‐*α*, IL‐1*β* and pro‐apoptotic proteins Bax, C‐caspase‐3, and anti‐apoptotic protein Bcl‐2 in BV‐2 microglia cells was significantly up‐regulated after LPS induction. When treated with scutellarin, the expression levels of p‐PI3K, p‐AKT, p‐GSK3*β*, and anti‐apoptotic protein Bcl‐2 were further up‐regulated, whereas the expression of p‐NF‐*κ*B, TNF‐*α*, IL‐1*β*, and pro‐apoptotic proteins Bax and C‐caspase‐3 was inhibited. It is concluded that the effect of scutellarin on microglia‐mediated inflammation and apoptosis in LPS‐induced MCAO rats may be partially dependent on the PI3K/AKT/GSK3*β* related mechanism and promoting the activation of the PI3K/AKT/GSK3*β* signaling pathway may provide a therapeutic approach to inhibit neuroinflammatory injury and apoptosis. The results of the Western blotting and fluorescence analysis at 3 days in vivo were consistent with those of the in vitro cell experiments.

The present study observed a significant reduction in the expression of p‐PI3K, p‐AKT, and p‐GSK3*β* after treatment with the PI3K pathway inhibitor LY294002 combined with scutellarin, implying that LY294002 could effectively inhibit activation of this pathway. Concurrently, there was a significant increase in the expression of p‐NF‐*κ*B, inflammatory factors TNF‐*α* and IL‐1*β*, and pro‐apoptotic proteins, Bax and C‐caspase‐3. In contrast, the expression of the anti‐apoptotic protein Bcl‐2 decreased, suggesting that scutellarin may regulate microglia‐mediated inflammation and apoptosis by promoting the activation of the PI3K/AKT/GSK3β signaling pathway and inhibiting the activation of the NF‐*κ*B pathway.

## 5 CONCLUSION

**FIGURE 12:**
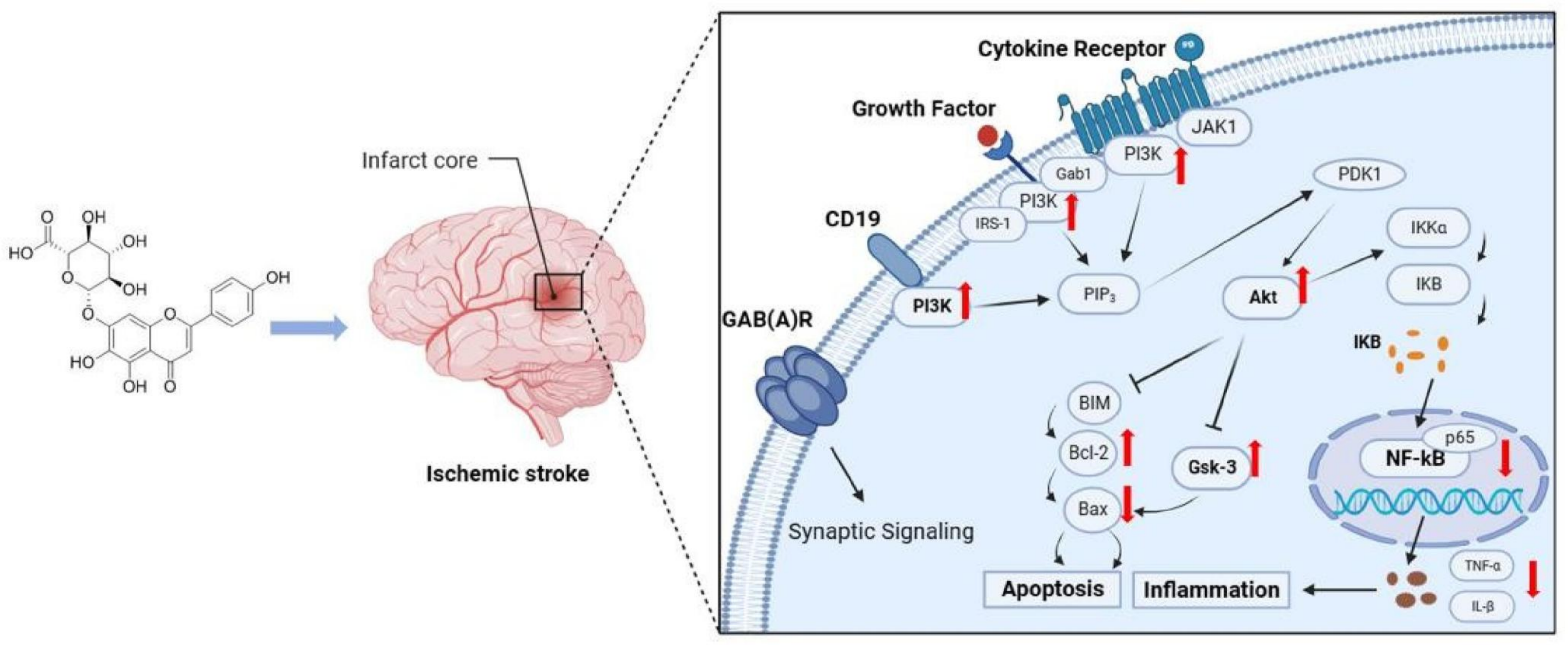
Schematic diagram illustrating the regulatory mechanism of scutellarin in microglia‐mediated neuroinflammation and apoptosis in IS. Scutellarin inhibited the NF‐*κ*B pathway, pro‐apoptotic proteins Bax and caspase‐3, and inflammatory factors TNF‐*α* and IL‐1*β* in microglia after IS. It also promoted the activation of the PI3K/AKT/GSK3β pathway and anti‐apoptotic protein Bcl‐2. Conversely, the PI3K pathway inhibitor promoted the expression of the NF‐*κ*B pathway, pro‐apoptotic proteins, and inflammatory factors while inhibiting the expression of the PI3K/AKT/GSK3*β* pathway and anti‐apoptotic protein Bcl‐2. These results suggest that scutellarin regulates activated microglia‐mediated neuroinflammation and apoptosis via the PI3K/AKT/GSK3*β* and NF‐*κ*B pathways.

In conclusion, PI3K/AKT/GSK3β, the NF‐*κ*B signaling pathway, neuroinflammatory factors, and apoptotic proteins were activated in LPS‐induced BV‐2 microglia and cerebral ischemia‐activated microglia. Scutellarin significantly reduced the expression of the NF‐*κ*B signaling pathway, inflammatory factors, and pro‐apoptotic proteins. Moreover, it promotes the expression of PI3K/AKT/GSK3*β* and anti‐apoptotic proteins. The PI3K inhibitor LY294002 was used to confirm that scutellarin inhibits microglia‐mediated inflammation and apoptosis, activates the PI3K/AKT/GSK3*β* signaling pathway, inhibits the activation of the NF‐*κ*B pathway, and thus, plays an anti‐inflammatory and anti‐apoptotic role in neuroprotection (Figure 12).

## AUTHOR CONTRIBUTIONS

Zhaoda Duan: Conceive and conduct experiments, collate and analyze data, and write the original draft articles. Wei Miao and Jing He: Cell culture and model replication. Haolun Chen and Dongyao Xu: Experimental data collection and sorting. Zhi Qi and Li Yang: Animal rearing was replicated with the MCAO model. Chunyun Wu and Wenji Jia: Fund support, experimental guidance, and manuscript revision.

## CONFLICT OF INTEREST STATEMENT

The authors declare that they have no competing interests.

## ETHICS STATEMENT

All animal experimental and animal use protocols were approved by the Experimental Animal Ethics Committee of Kunming Medical University (approval number: kmmu20220066).

## Data Availability

The authors do not have permission to share data.
